# Recombinase polymerase amplification assay combined with a dipstick-readout for rapid detection of *Mycoplasma ovipneumoniae* infections

**DOI:** 10.1371/journal.pone.0246573

**Published:** 2021-02-04

**Authors:** Sandeep K. Gupta, Qing Deng, Tanushree B. Gupta, Paul Maclean, Joerg Jores, Axel Heiser, D. Neil Wedlock

**Affiliations:** 1 Animal Health, AgResearch, Hopkirk Research Institute, Grasslands Research Centre, Palmerston North, New Zealand; 2 Food Safety & Assurance, AgResearch, Hopkirk Research Institute, Grasslands Research Centre, Palmerston North, New Zealand; 3 Bioinformatics and Statistics, AgResearch, Grasslands Research Centre, Palmerston North, New Zealand; 4 Institute of Veterinary Bacteriology, University of Bern, Bern, Switzerland; Defense Threat Reduction Agency, UNITED STATES

## Abstract

*Mycoplasma ovipneumoniae* infects both sheep and goats causing pneumonia resulting in considerable economic losses worldwide. Current diagnosis methods such as bacteriological culture, serology, and PCR are time consuming and require sophisticated laboratory setups. Here we report the development of two rapid, specific and sensitive assays; an isothermal DNA amplification using recombinase polymerase amplification (RPA) and a real-time PCR for the detection of *M*. *ovipneumoniae*. The target for both assays is a specific region of gene WP_069098309.1, which encodes a hypothetical protein and is conserved in the genome sequences of ten publicly available *M*. *ovipneumoniae* strains. The RPA assay performed well at 39°C for 20 min and was combined with a lateral flow dipstick (RPA-LFD) for easy visualization of the amplicons. The detection limit of the RPA-LFD assay was nine genome copies of *M*. *ovipneumoniae* per reaction and was comparable to sensitivity of the real-time PCR assay. Both assays showed no cross-reaction with 38 other ovine and caprine pathogenic microorganisms and two parasites of ruminants, demonstrating a high degree of specificity. The assays were validated using bronchoalveolar lavage fluid and nasal swab samples collected from sheep. The positive rate of RPA-LFD (97.4%) was higher than the real-time PCR (95.8%) with DNA as a template purified from the clinical samples. The RPA assay was significantly better at detecting *M*. *ovipneumoniae* in clinical samples compared to the real-time PCR when DNA extraction was omitted (50% and 34.4% positive rate for RPA-LFD and real-time PCR respectively). The RPA-LFD developed here allows easy and rapid detection of *M*. *ovipneumoniae* infection without DNA extraction, suggesting its potential as a point-of-care test for field settings.

## Introduction

Bronchopneumonia is a multifactorial disease that involves interactions between different bacterial and viral pathogens as well as predisposing factors such as immunocompromised hosts, environmental factors and stress [1–3]. Because of this complexity and multifactorial nature, sheep pneumonia is commonly known as ovine respiratory disease complex and includes *Mannheimia haemolytica*, *Mycoplasma ovipneumoniae* and Parainfluenza virus type 3 [4, 5]. Pneumonia generally results in sudden death or a long, drawn-out illness both causing considerable economic losses to sheep industries worldwide. In addition, pneumonia is a major animal welfare concern and economically there are impacts associated with lower growth rates, downgrading and condemnations of carcasses and treatment and prevention costs [6, 7]. The annual average cost of pneumonia to the NZ sheep industry between $32M and $79M, excluding the cost of animal deaths [8].

While a wide variety of microorganisms have been reported in the lungs of sheep [9], mycoplasma species are associated with upper respiratory tract infections and can lead to onset of pneumonia in sheep [10, 11]. Mycoplasmas primarily infect animals that are under stress due to environmental factors such as cold, heat or dense housing. This results in subclinical interstitial bronchopneumonia that often predisposes the lower respiratory tract to other secondary infections with pathogens such as *M*. *haemolytica* and Parainfluenza virus type 3 leading to chronic pneumonia [12]. Traditional microbiological techniques for diagnosis of *M*. *ovipneumoniae* are labour-intensive and time consuming. Usually up to two weeks are needed to grow the bacteria due to its slow-growing nature [13]. While various PCR based methods have been developed for diagnosis and epidemiological studies of *M*. *ovipneumoniae* infection in sheep [14, 15], they require sophisticated instrumentation, time and trained personnel. A loop-mediated isothermal amplification (LAMP) method has been developed to detect *M*. *ovipneumoniae* [16]. LAMP assays are difficult to develop needing 6–8 primers and require specialised commercial software packages for primer design. In addition, both PCR and LAMP assays require high quality purified DNA. These technical challenges have hindered the use of these methods as field diagnostic tools.

Recombinase Polymerase Amplification (RPA) assays work at isothermal temperatures between 25°C to 42°C, in which the target DNA can be amplified within 20 min from a wide variety of organisms. The amplified product can be visualized using various methods such as fluorescence or a lateral flow type dipstick. One of the advantages of RPA is that it is tolerant to numerous substances, which inhibit amplification in PCR-based assays [17]. RPA can amplify target nucleic-acid in different samples including plasma, sputum/respiratory washes, and pleural fluid [18, 19]. This is of a particular importance for RPA-based pen-side diagnostic tests because impure samples can be tested quickly without the need for nucleic acid extraction.

In this study, we describe the development of RPA with an nfo-probe combined with a lateral flow dipstick (RPA-LFD) assay for rapid and sensitive detection of *M*. *ovipneumoniae*. For comparative purposes, a real-time PCR assay was also developed to detect *M*. *ovipneumoniae*. The performance parameters of the two assays were compared using sheep clinical samples with or without a prior DNA extraction step.

## Materials and methods

### Strains and clinical samples

The *M*. *ovipneumoniae* reference strain (Accession number: 1959) was obtained from the New Zealand Reference Culture Collection: Medical Section (NZRM) and was used for the optimization and evaluation of real-time PCR and RPA. Three other *M*. *ovipneumoniae* isolates (16, 90 and 103) were isolated from New Zealand sheep with pneumonia [20]. Fifteen additional Mycoplasma strains representing 10 species and sub-species were used to evaluate the specificity of real-time PCR and RPA (S1 Table). All the strains were cultured in Mycoplasma liquid medium (0.3 yeast extract, 20% horse serum, 0.025% thallium acetate, 500 U/mL penicillin and 0.1% glucose and phenol red) at 37°C for 24–72 h until a colour change from red to orange was observed. Non-Mycoplasma strains were also obtained from the NZRM and were grown as recommended (S1 Table).

Clinical samples used in the study were collected as part of other field trials conducted by the team and Animal ethics approval was obtained by the AgResearch Grassland’s Animal Ethics Committee, Palmerston North, New Zealand for all procedures involving animals. A total of 192 samples were used in the study comprising 142 samples of bronchoalveolar lavage fluid (BALF), and nasal swabs from sheep (n = 25 each) obtained before and after experimental infection with *M*. *ovipneumoniae* and *M*. *haemolytica*.

### DNA extraction

Genomic DNA was extracted from pure cultures using a Quick DNA Fungal/Bacterial Kit according to the manufacturer’s instructions (Zymo Research, CA, USA). The purified DNA was treated with RNaseA to remove RNA contamination using a previously described method [21]. Genomic DNA preparations from all bacterial species were confirmed via 16S rRNA gene amplification and Sanger sequencing.

### Primer and probe design for RPA and real-time PCR

The full genome sequences of *M*. *ovipneumoniae* (NZ_JOTK01000063, NZ_JOTF01000020, NZ_KV765928, NZ_KV766053, NZ_AGRE01000005, NZ_KV765945, NZ_JOTI01000061, NZ_JOTE01000019, NZ_JFAD01000009, NZ_JAKV01000002) were used to design primers and probe against the WP_069098309.1 gene according to TwistDX guidelines (TwistDx, United Kingdom). Both forward and reverse primers were 30–35 nucleotide (nt) long and the reverse primer was modified with a biotin tag at the 5’ end. The RPA-nfo probe was 45 nt long and contained fluorophore 6-carboxyfluorescein (6-FAM) at the 5’ end, an internal abasic tetrahydrofuran spacer (THF) and a polymerase extension blocking group (C3-spacer) modifications. All the primers and probe for RPA were synthesized by Integrated DNA Technologies (USA) and purified by HPLC. For real-time PCR, primers were designed with the Geneious Software version 2019.1.1 [22] and synthesised by Integrated DNA Technologies (USA). *In silico* specificity of the primers and probe was determined using the pattern searching tool fuzznuc from the EMBOSS package [23] against selected bacterial genomes (S2 Table).

### Generation of DNA standard plasmid

The WP_069098309.1 fragment (280 bp) was synthesised by GeneScript and cloned into pCR-TOPO vector (ThermoFisher Scientific, New Zealand). The plasmid containing the WP_069098309.1 fragment was transformed into *E*. *coli* DH5-α cells by heat-shock and positive clones were selected using kanamycin. The standard DNA with WP_069098309.1 target sequence was extracted using PureYield Plasmid Midiprep purification kit (Promega, WI, USA) and quantified using Qubit fluorometer according to the manufacturer’s instructions (ThermoFisher Scientific, New Zealand). The DNA copy number was calculated based on the equation: DNA copy number = (M × 6.02 × 10^23^ × 10^−9^)/(n × 660)^28^, M: molecular weight, n: plasmid concentration measured at 260 nm. The DNA standards were prepared as 10^7^, 10^6^, 10^5^, 10^4^, 10^3^, 500, 250, 100, 10 copies/μL and stored in aliquots at −20°C until used.

### SYBER green real-time PCR for amplification of WP_069098309.1 gene

Real-time PCR was performed using SYBR® Premix Ex Taq™ II (TliRNaseH Plus) reagents (Takara BioInc, Japan) according to the manufacturer’s instructions. The reaction was carried out as described previously [24]. Briefly, each reaction was carried out in a 10 μL volume containing 5 μL of 2 × SYBR Premix Ex Taq II, 0.3 μL of each forward and reverse primers (10 μM), 1 μL of DNA and 3.4 μL nuclease-free water. The real-time PCR program comprised initial denaturation for 3 min, followed by 40 cycles of 95°C for 10 s, 60°C for 30 s. Amplification efficiencies for the real-time PCR reactions were between 1.6 and 1.8. Each sample was measured in duplicate. Melt curve analysis showed that positive samples produced a single discrete peak for the primer pair, indicating that the reaction product contained a single amplicon.

### RPA-LFD assay

RPA reactions were performed according to the manufacturer’s instructions (TwistAmp nfo kit, United Kingdom). A typical 50 μL RPA reaction contained 29.5 μL of rehydration buffer, 14.4 μL nuclease-free water, 2.0 μL each of a forward primer/reverse primer (10 mM), 0.6 μL probe (10 mM) and 1.0 μL of template. Finally, 2.5 μL magnesium acetate (280 mM) was added to each reaction followed by a brief centrifugation. The tubes were incubated at 39°C in a thermocycler for 20 min. According to the TwistDx recommendations, the samples were mixed 6–8 times after 4 min incubation followed by additional incubation for 16 min. For visualization of amplicons on agarose gel, the RPA products were purified using a PCR purification kit (Promega, WI, USA) and detected by electrophoresis on a 2% agarose gel.

As an alternative to electrophoresis, the dual-labelled amplicons produced by the RPA-nfo reaction were visualized using LFD. Briefly, 2 μL of the RPA-nfo reaction was diluted in 98 μL of PBS Tween buffer. A 10 μL portion of this mixture was applied onto the sample pad of LFD (HybriDetect, Milenia Biotec GmbH, Germany) and the LFD was vertically placed into 50 μL PBS Tween buffer for 2 min. Photographs were taken with a Samsung camera (A5 2017).

### RPA reaction conditions and parameter optimisation

In order to achieve optimal performance of primers and probe for the RPA, several primers were screened using the standard DNA containing the WP_069098309.1 gene. Next, temperature and time were examined for the optimal performance of RPA reaction using the selected primers and LF-probe. RPA reaction was conducted at temperatures ranging between 20–45°C and for times ranging between 5–30 min.

### Determination of specificity and sensitivity of the RPA-LFD assay

The specificity of the *M*. *ovipneumoniae* RPA assay was determined using genomic DNA from bacterial and mammalian species listed in S1 Table. Each RPA reaction contained genomic DNA corresponding to 1 x 10^2^ genome copies of the strain tested. Genomic DNA of *M*. *ovipneumonaie* was included in each run as a positive control. The sensitivity of the RPA was determined using serially diluted genomic DNA and the standard ranging from 1 ng to 10 fg and 10^7^ to 10^1^ copies per reaction, respectively.

### Assessment of RPA-LFD using bronchoalveolar lavage fluid and nasal swab samples

Bronchoalveolar lavage fluid (BALF) samples were collected from sheep (n = 142) from a slaughterhouse in the Manawatu region, New Zealand. Lungs were collected from the animals and 50 mL of saline was poured into the trachea and the lung wash was collected into a 50 mL tube and stored on ice for transportation back to the laboratory. In the laboratory, 2 mL BALF was transferred into a tube and centrifuged for 5 min at 5,000 × *g* at 4°C and the supernatant was discarded. The pellet was re-suspended in 200 μL of PBS and divided into two equal parts. DNA was isolated from one part using Quick-DNA 96 Plus kit (Zymo Research, CA, USA) and the second part was subjected to heat lysis by incubating at 100°C for 15 min in a thermocycler. The isolated DNA and lysate from the clinical samples were stored at − 20°C until further use.

Ability of the RPA to detect *M*. *ovipeumonaie* in sheep was also determined using nasal secretions from animals (n = 25 each) before and after experimentally challenging with *M*. *ovipneumoniae* and *M*. *haemolytica*. Briefly, nasal secretions were collected from the animals by inserting and rubbing a cotton swab into the nostrils. The swab was then immersed in 1 mL of PBS and immediately stored at 4°C. The swab was squeezed against the wall of the tube and centrifuged for 5 min at 5,000 × *g* at 4°C and the supernatant was discarded. The pellet was re-suspended in 200 μL of PBS and divided into two equal parts for DNA isolation and lysate preparation as mentioned above.

A total of 1 μL of the isolated DNA and lysate was used as a template in a final reaction volume of 50 μL to perform the RPAs. The RPA reactions were performed in duplicate.

### Statistical analysis

Microsoft Office Excel software was used to perform statistical analysis. Differences between the real-time and RPA-LFD performance were analyzed with the Fisher Exact Test. Differences were considered significant when a P value of < 0.05 was obtained.

## Results

### Identification of WP_069098309.1 gene in *M*. *ovipneumoniae*

In order to identify a gene unique to *M*. *ovipneumoniae*, genomes from 36 pathogenic bacteria, and two parasites of ruminants, bovine and ovine (S2 Table), were downloaded from NCBI and added to a BLAST+ version 2.7.1 nucleotide database [25]. Protein sequences from a total of 10 publicly available genome sequences of *M*. *ovipneumoniae* were searched against the 40 genomes using the “blastx” command from BLAST+. A perl script was used to extract *M*. *ovipneumoniae* proteins that did not align to any of the non-*M*. *ovipneumoniae* genomes. These extracted proteins were searched against the 10 *M*. *ovipneumoniae* genomes using “blastx” function within Geneious prime version 2019.1.1 [22] with an identity threshold of 90% an expect value cut off of 1e-50 to find unique, single-copy, genes belonging only to *M*. *ovipneumoniae* genomes. This resulted in identification of WP_069098309.1 gene coding for a hypothetical protein, which was again searched against the *M*. *ovipneumoniae* genomes to ensure uniqueness and copy number of the gene in the species.

### Design and optimization of real-time PCR for WP_069098309.1 gene and detection limit of real-time PCR

The WP_069098309.1 genes from all ten *M*. *ovipneumoniae* genomes were aligned and a 350 bp long region was selected to design primers for real-time PCR (Table 1). PCR annealing temperature was optimized and found to be between 55°C and 60°C, which resulted in amplification of a single amplicon with an expected size of 199 bp (S1 Fig). The results also showed that PCR for WP_069098309.1 gene produced a much brighter amplicon compared to previously reported PCR for *p113* gene [14] as visualized on an agarose gel at all the annealing temperatures tested (S1 Fig).

**Table 1:** Primers and probe used in RPA-LFD assay and real-time PCR development.

Primer/probe name	Oligonucleotide sequences (5′-3′)	Genome location (NZ_JOTE01000019)	Amplification size (bp)
M.ovi_RPA-F1	TATCGCAAAATATAAAGAAGATTTTCACAAAA	15,018–14,987	342
M.ovi_RPA-F2	TGGTTGAAAAGGCACCTAAAATTAGCAC	14,980–14,953	304
M.ovi_RPA-F3	TGAGTATTCCAAGAATTCCTTTTAGTGCC	14,902–14,873	226
M.ovi_RPA-F4	CTTTTAGTGCCAATTTAACTTTATTTGATCAT	14,884–14,853	208
M.ovi_RPA-R	Biotin-GTTGTCAGTATGTTAATAACGATTTGCCA	14,677–14,705	
M.ovi_RPA-LF probe	FAM-ATCAATTTTAATTTATGGTCAACTTTAGTG(THF)TCATGCCTTACTTAG-C3	14,792–14,747	116
M.ovi_real-time PCR-F	CCAAGAATTCCTTTTAGTGCCAA	14,894–14,872	199
M.ovi_real-time PCR-R	CGATTTGCCACAAATAAAGTC	14,716–14,696	

**Fig 1:**
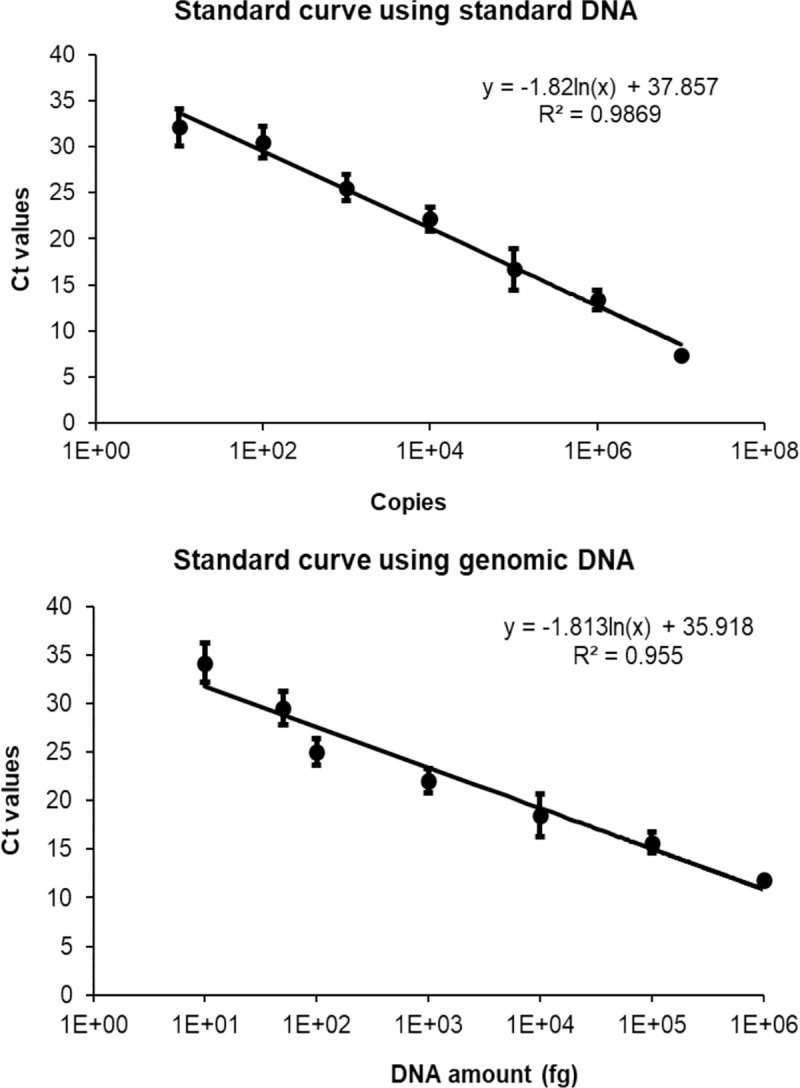
Detection limit of real-time PCR for WP_069098309.1 gene using pDNA and genomic DNA as templates. (A), Real-time PCR using serially diluted standard DNA standard with 10^7^ to 10^1^ copies per reaction; (B), Real-time PCR using serially diluted genomic DNA from 1 ng to 10 fg per reaction.

The detection limit of real-time PCR was evaluated using the standard DNA containing the WP_069098309.1 target sequence and genomic DNA isolated from *M*. *ovipneumoniae*. Using serially diluted standard DNA as a template, the detection limit of the real-time PCR assay was found to be 1 x 10^2^ copies per PCR reaction, while detection limit of real-time PCR using genomic DNA as a template was 10 femtogram (Fig 1). These results indicate that real-time PCR for WP_069098309.1 gene was highly sensitive.

### Design and optimization of RPA primers and probe and their specificity *in silico*

Four candidate forward primers with a single biotin-labelled reverse primer (BR) and an nfo-probe (LFP) were designed to amplify a region of the WP_069098309.1 gene (Table 1). In order to ensure that the sequences of the primers and the probe were unique to target this single-copy gene in *M*. *ovipneumniae*, the selected primers and probe were screened against the genomes (bacterial, parasites, bovine and ovine) using an *in-silico* approach (S2 Table). Our results indicated that no complementary regions were found when up to 5 nucleotide mismatches were allowed in the analysis for the forward and reverse primers as well as the probe. However, allowing 10 nucleotide mismatches gave rise to matches to both the primers but not the probe. The binding positions of the primers were located a minimum of 10kb apart on bacterial genomes and unlikely to amplify a product, which suggested specificity of the primers to the target gene of *M*. *ovipneumniae*.

**Fig 2:**
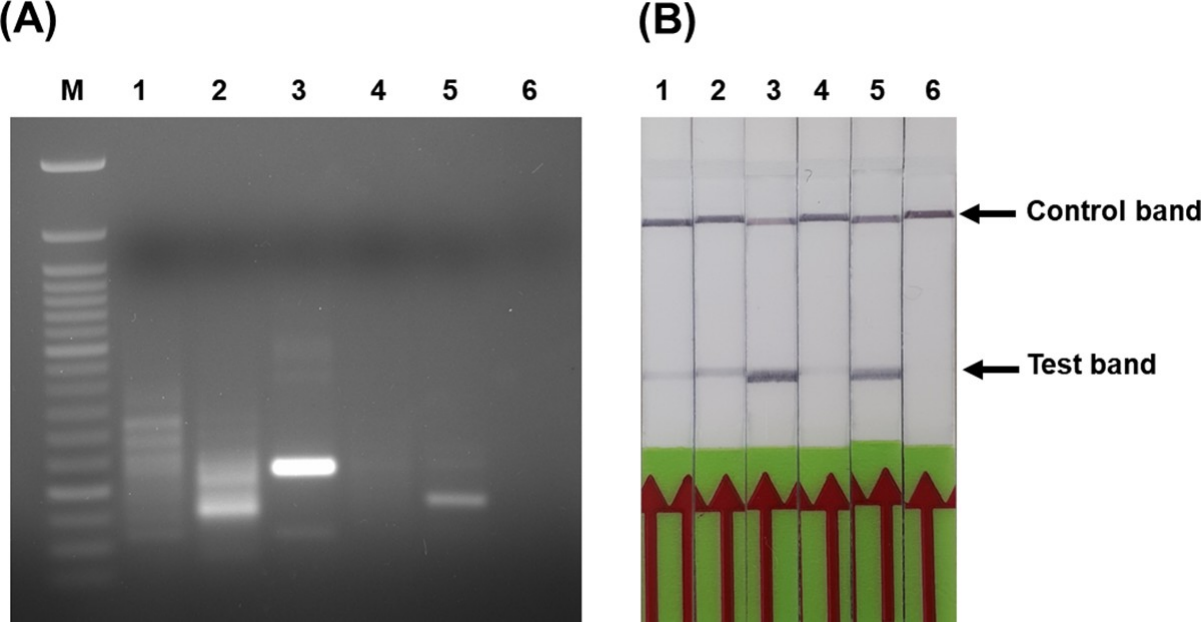
Screening of primers and nfo-probe for *M*. *ovipneumoniae* RPA assay. (A), RPA amplified products using four candidate forward primers with a single biotinylated reverse primer (BR) and a probe (LFP). Lane M, represents 50-base pair molecular weight ladder, 1; F1/BR/LFP, 2; F2/BR/LFP, 3; F3/BR/LFP, 4; F4/BR/LFP, 5; positive control (supplied by Twist Amp nfo kit), 6; negative control (DNase-free water). (B), RPA-LFD analysis of dual-labelled products generated using the designed primers and probe. Lane 1; F1/BR/LFP, 2; F2/BR/LFP, 3; F3/BR/LFP, 4; F4/BR/LFP, 5; positive control (supplied by Twist Amp nfo kit), 6; negative control (DNase-free water).

The *in-silico* analysis confirmed that the primers and the probe fulfilled the requirements of specific RPA. Based on the *in-silico* findings, four candidate forward primers with a single biotin-labelled reverse primer (BR) and an nfo-probe (LFP) combination targeting WP_069098309.1 gene were screened against the purified genomic DNA from *M*. *ovipneumniae* using a TwistAmp nfo reaction. Initially, the ability of the primers and the probe combination to amplify specifically labelled amplicons was analysed using agarose gel electrophoresis. The results showed that M.ovi_RPA-F3 and M.ovi_RPA-R primers and the probe were identified as capable of amplifying a 226 bp size product with great efficiency (Fig 2A). Therefore, the F3/BR/LFP set were chosen for subsequent evaluation. The F3/BR/LFP set were subjected to the RPA assay using TwistAmp™ nfo reagents followed by running the dual-labelled amplicons on a LFD. The results showed that the F3/BR/LF set produced the most efficient amplification as indicated by a test line within 2 min on the LFD (Fig 2B).

### Evaluation of amplification temperature and time

**Fig 3:**
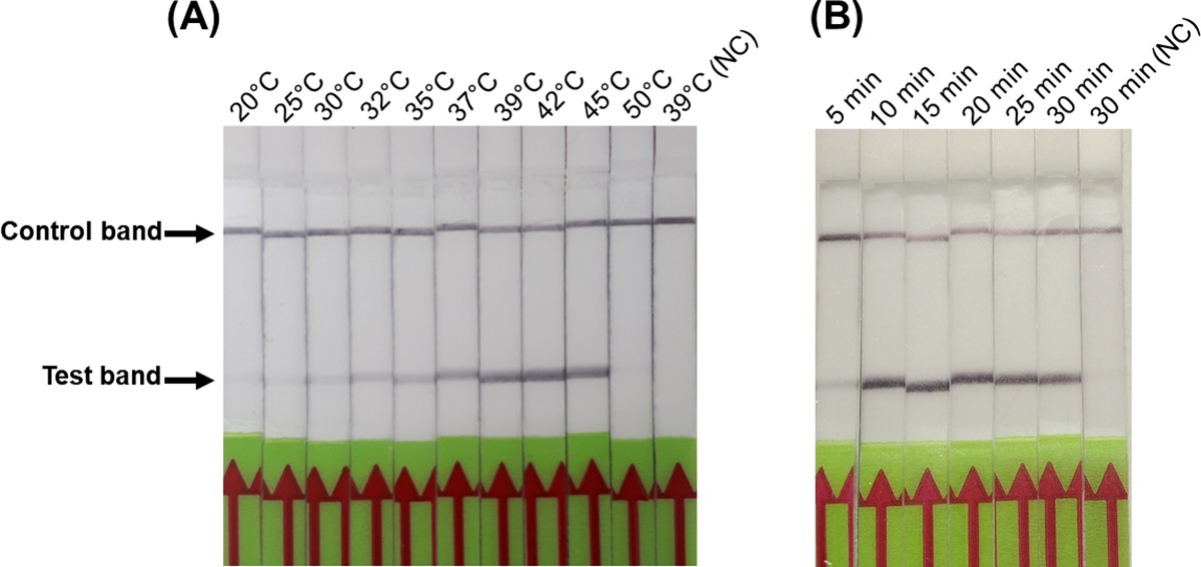
Optimization of temperature and time for *M*. *ovipneumoniae* RPA-LFD assay. (A), The RPA amplification was performed at temperatures ranging between 20°C and 50°C and was operative between 30°C and 45°C. (B), Evaluation of amplification time for the RPA assay where the amplification was performed for various time ranging from 5 to 30 min at 39°C. Amplified dual-labelled amplicons were run on LFD and the positive signal be detected as early as 2 min. Negative control (water) did not produce any amplification.

In order to determine the optimal temperature for amplification, the RPA reaction was conducted at temperatures ranging between 20°C to 50°C. The results showed that the RPA can be performed at various temperatures ranging from 30°C to 45°C. The brightness of the test bands changed with temperature, but the intensity of the test band was maximum between 37°C to 42°C, indicating this temperature range was optimal for the RPA assay (Fig 3A). Next, we examined the optimal time for the RPA assay by running the RPA amplification between 5 and 30 min. The results indicated that the test band was visible as early as 5 min after the amplification, but the test band was brightest between 10 min and 30 min (Fig 3B). Based on these results, all the RPA reactions were conducted at 39°C for 20 min.

### Sensitivity of the RPA-LFD

**Fig 4:**
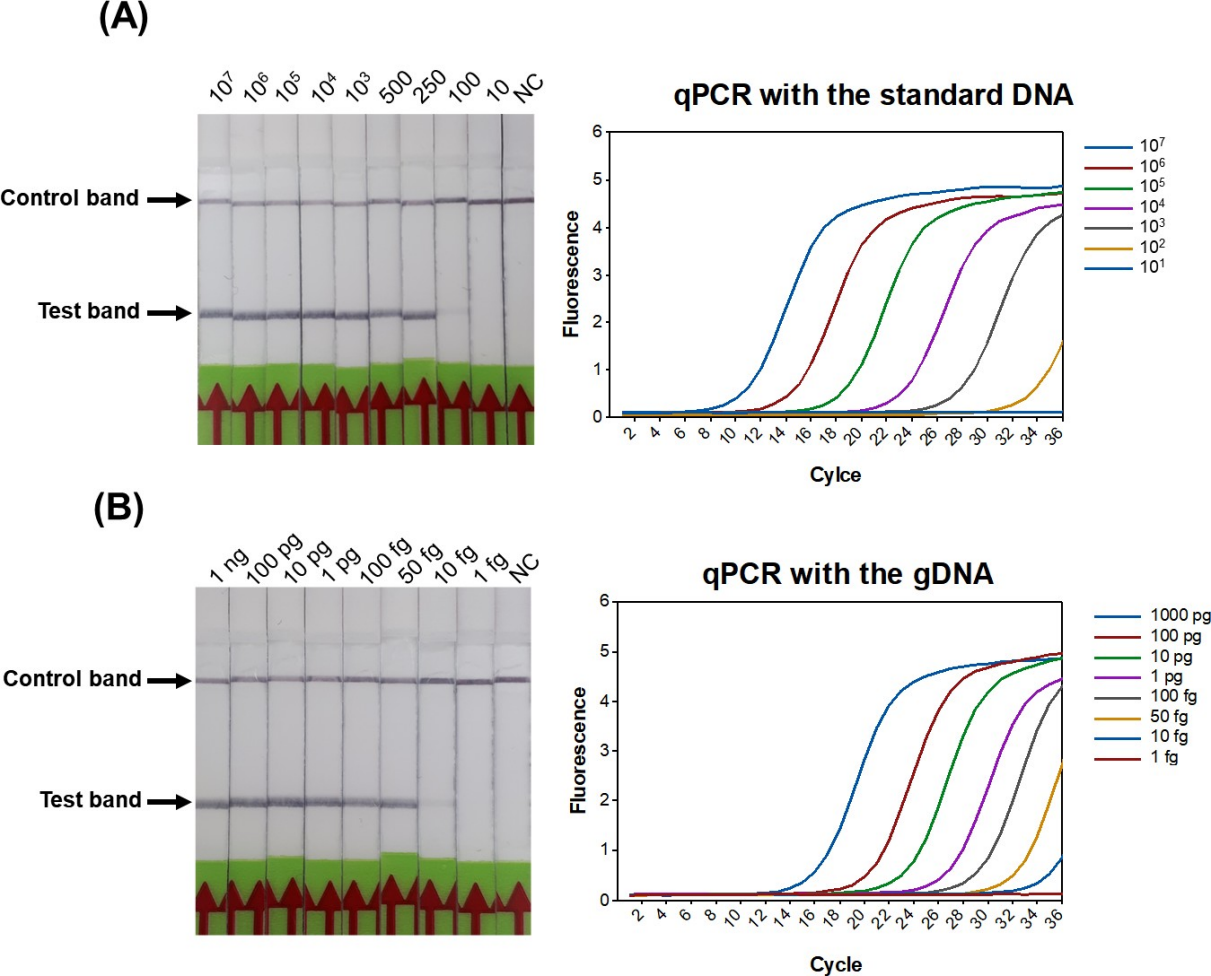
Comparison of the sensitivities of the RPA-LFD and real-time PCR. Molecular sensitivity of two methods was determined using serially diluted standard DNA and genomic DNA of *M*. *ovipneumoniae*. (A) Results of the RPA-LFD and real-time PCR using standard DNA as template showed a detection limit of 100 copies per reaction. (B) Results of the RPA-LFD and real-time PCR using genomic DNA of *M*. *ovipneumoniae* as template showed detection limit of 10 fg per reaction. NC; negative control (DNase-free water).

The analytical sensitivity of the RPA-LFD was assessed using the standard DNA and genomic DNA purified from *M*. *ovipneumoniae* and compared with that of real-time PCR developed for the WP_069098309.1 gene. The results showed that the sensitivity of the RPA-LFD assay for the standard DNA was 100 copies per reaction, which was similar to the real-time PCR assay (Fig 4A). The RPA-LFD assay could detect as low as 10 fg of *M*. *ovipneumoniae* genomic DNA, which is equivalent to 9 genome copies per reaction (Fig 4B). The sensitivity of the RPA-LFD assay was similar to that of the real-time PCR assay in detecting *M*. *ovipneumoniae* genomic DNA, which also showed a detection limit of 10 fg.

### Specificity of RPA-LFD and real-time PCR

Specificity of the RPA-LFD assay was evaluated using purified genomic DNA from various organisms including Mycoplasmas and common respiratory tract pathogens, other pathogens of ovine and caprine species, parasites, bovine and ovine genomic DNA. As expected, the results showed that the test band was only visible in the presence of *M*. *ovipneumoniae* DNA and was negative for DNA from all of the 40 other species tested. These results indicated a high specificity of the RPA-LFD towards *M*. *ovipneumoniae* and showed no cross-reaction against any of the organisms tested (Table 2, S2 Fig).

**Table 2:** Specificity of *M*. *ovipneumoniae* RPA-LFD assay against other organisms.

Species	RPA	Real-time PCR
*Mycoplasma ovipneumoniae* reference strain: 1959	Positive	Positive
*Mycoplasma ovipneumoniae* isolate 16	Positive	Positive
*Mycoplasma ovipneumoniae* isolate 90	Positive	Positive
*Mycoplasma ovipneumoniae* isolate 103	Positive	Positive
*Mycoplasma bovis* (PG45)	Negative	Negative
*Mycoplasma dispar*	Negative	Negative
*Mycoplasma bovirhinis* (17D0278)	Negative	Negative
*Mycoplasma bovoculi*	Negative	Negative
*Mycoplasma bovigenitalium*	Negative	Negative
*Mycoplasma canis*	Negative	Negative
*Mycoplasma gallinarum*	Negative	Negative
*Mycoplasma putrefaciens*	Negative	Negative
*Mycoplasma capricolum*	Negative	Negative
*Mycoplasma mycoides *subsp.* capri*	Negative	Negative
*Mycoplasma feriruminatoris*	Negative	Negative
*Mycoplasma leachii*	Negative	Negative
*Mannheimia haemolytica*	Negative	Negative
*Pasteurella multocida*	Negative	Negative
*Staphylococcus aureus*	Negative	Negative
*Streptococcus uberus*	Negative	Negative
*Streptococcus pyogenes*	Negative	Negative
*Streptococcus agalactiae*	Negative	Negative
*Salmonella typhimurium*	Negative	Negative
*Escherichia coli* O157:H7	Negative	Negative
*Enterobacter aerogenes*	Negative	Negative
*Pseudomonas aeruginosa*	Negative	Negative
*Bacillus subtilis*	Negative	Negative
*Bacillus cereus*	Negative	Negative
*Mycobacterium bovis*	Negative	Negative
*Mycobacterium paratuberculosis* K-10	Negative	Negative
*Mycobacterium paratuberculosis* C-type	Negative	Negative
*Mycobacterium paratuberculosis* S-type	Negative	Negative
*Clostridium tetani*	Negative	Negative
*Clostridium perfringens* Type C	Negative	Negative
*Clostridium novyi*	Negative	Negative
*Clostridium chauvoei*	Negative	Negative
*Clostridium septicum*	Negative	Negative
*Clostridium hemolyticum*	Negative	Negative
*Trueperella pyogenes*	Negative	Negative
*Listeria monocytogenes*	Negative	Negative
*Leptospira interrogans* serovar *Hardjo*	Negative	Negative
*Klebsiella pneumoniae*	Negative	Negative
*Haemonchus contortus*	Negative	Negative
*Teladorsagia circumcincta*	Negative	Negative
*Bos taurus*	Negative	Negative
*Ovis aries*	Negative	Negative

### Analysis of clinical samples

We compared two methods of sample preparation for the RPA assay. First the performance of the RPA-LFD was evaluated using 192 clinical samples collected from sheep. Of these, 142 BALF samples were collected from lungs at the abattoir in the Manawatu region, New Zealand and 50 nasal swabs were obtained before and after experimental infection of sheep with *M*. *ovipneumoniae* and *M*. *haemolytica*. The results showed that heat treatment of the samples at 100°C was sufficient to release DNA from mycoplasma in the sample. In a preliminary experiment, three different volumes of 1, 2.5 or 5 μL of lysed samples were tested in the RPA reaction. The results demonstrated that 1 μL of the sample as input material in the RPA reaction produced the brightest band on LFD compared to the 2.5 and 5 μL of the sample lysed sample (S3 Fig). The results indicated that 1 μL sample had minimal inhibitory effect on the RPA reaction, while a 2.5 or 5 μL sample had inhibitory effects on the RPA reaction. Therefore, a volume of 1 μL of the lysed clinical sample was used for the rest of the samples.

The RPA-LFD and real-time PCR were performed in parallel using the lysate and purified DNA. The results demonstrated that the RPA-LFD detected *M*. *ovipneumoniae* in higher numbers of BALF samples with 142 (100%) purified DNA and 65 (45.8%) lysate compared to the real-time PCR which detected *M*. *ovipneumoniae* in 140 (98.6%) BALF samples with purified DNA and 39 (38%) of lysate (Table 3). The RPA-LFD detected *M*. *ovipneumoniae* in 6 (24%) of the lysate samples prepared from nasal swabs and in 20 (80%) purified DNA samples (Table 3). In comparison, the real-time PCR detected *M*. *ovipneumoniae* in 19 (76%) purified DNA samples and in only 2 (8%) of the lysates prepared from nasal swab samples obtained before challenge of the sheep. All nasal swab lysate samples were positive for RPA-LFD and real-time PCR after challenge, indicating 100% positive rate of these assays. The results of the nasal swabs correlated with the presence of *M*. *ovipneumoniae* in lung lavages in experimentally challenged animals. All were positive for *M*. *ovipneumoniae* growth in BALF culture post challenge.

**Table 3:** Comparison of *M*. *ovipneumoniae* RPA-LFD and real-time PCR using clinical samples.

		RPA-LFD		Real-time PCR	
Sample	Number	Purified DNA	Lysate	Purified DNA	Lysate
BLAF from sheep	142	142 (100.0%)	65 (45.8%)	140 (98.6%)	39 (38%)
Nasal swab (BC*)	25	20 (80%)	6 (24%)	19 (76%)	2 (8%)
Nasal swab (AC**)	25	25 (100%)	25 (100%)	25 (100%)	25 (100%)
Total samples	192	187 (97.4%)	96 (50.0%)	184 (95.8%)	66 (34.4%)

*BC: Nasal swab collected from sheep before experimental challenge with *M*. *ovipneumoniae* and *M*. *haemolytica*

**AC: Nasal swab collected from sheep after experimental challenge with *M*. *ovipneumoniae* and *M*. *haemolytica*

Overall, the RPA-LFD assay showed a detection rate of 50% when lysate was used as the input material and was significantly better (*p* < 0.01) compared to a detection rate of 34.4% for the real-time PCR. The detection rates were 97.4% and 95.8% using purified DNA as template with RPA-LFD and real-time PCR, respectively (Table 3). These results suggested that the lysate can be successfully used as input material for detection of *M*. *ovipneumoniae* in a RPA-LFD based assay.

## Discussion

Testing of livestock for diseases is an important part of farm management worldwide. In recent years, the focus of diagnostic assays has shifted towards fast, easy and molecular-based detection methods compared to the conventional and time-consuming methods such as pathogen culture or ELISA, which are not suitable for point-of-care testing. In this study, we developed new highly sensitive and specific RPA-LFD and real-time PCR assays for diagnosis of *M*. *ovipneumoniae* with a detection limit of 10 fg (9 genome equivalent). Although the RPA-LFD for *M*. *ovipenumoniae* developed here is not ideal for quantitative analysis of nucleic acid, important features of the assay such as its simple set up, speed, sensitivity, no prior DNA extraction, and the ability to visualise the results with the naked eyes make this assay attractive as a diagnostic test. The entire assay can be performed within 25 min and requires only a constant temperature of 39°C with minimum equipment, making this assay an attractive option to develop into a pen-side point-of-care diagnostic test for farm settings.

RPA is a relatively new isothermal amplification method that can successfully amplify target DNA in less time and at lower temperatures compared to other amplification methods such as PCR and LAMP. The latter require expensive equipment and trained staff. Previously, a real-time PCR based method has been described for *M*. *ovipneumoniae*, which amplifies *p113* gene with a detection limit of 220 genome copies [14]. However, PCR for the WP_069098309.1 gene developed in this study, resulted in a higher intensity amplicon compared to the PCR for *p113*, suggesting higher sensitivity and better diagnostic potential of WP_069098309.1 gene compared to the previously reported *p113* gene of *M*. *ovipneumoniae* [14]. In addition, a recent study described a LAMP-based diagnostic assay for *M*. *ovipneumoniae* with a detection limit of 100 CFU/mL [16]. Although LAMP is also an isothermal based amplification, it requires a longer assay time (45–60 min), higher temperature (60–65°C), more complex primers and DNA extraction to efficiently detect the target DNA. PCR is still considered as one of the best methods for nucleic acid amplification-based detection in laboratories and often used to compare performance of newly developed amplification-based methods. In the current study, we demonstrated that the sensitivity and specificity of a newly developed real-time PCR assay was comparable to that of the RPA-LFD, providing choice of two assays to be used in different settings for detection of *M*. *ovipneumoniae*. RPA-LFD could be useful where rapid detection of *M*. *ovipneumoniae* is required with presence or absence type analysis, while real-time PCR will be suitable for quantitative detection of *M*. *ovipneumoniae*.

RPA-LFD is an attractive method for rapid and accurate visual diagnosis in the field with limited resources [18, 19]. An additional advantage is the availability of the RPA reaction components in lyophilised format, which are stable at ambient temperature for at least 6 months, while the reagents for all the other nucleic acid-based detection techniques require refrigeration. This makes RPA a highly suited technology to implement as a point-of-care methodology in the field. Another advantage of RPA-based assays is that they can tolerate common inhibitors of conventional PCR. RPA has been shown to work with nucleic acid extracted from blood, serum, faeces, urine, and milk [26–31]. RPA has also been shown to amplify target DNA present in faeces, and pleural fluid without DNA extraction [32, 33]. The results presented here are in agreement with previous reports and show that the RPA-LFD assay can detect *M*. *ovipneumoniae* in BALF and nasal secretions after heat lysis, which was significantly better compared to the real-time PCR assay. This is of importance for RPA based pen-side diagnostic test because impure samples can quickly be tested without the need for nucleic acid extraction. The RPA-LFD method described here requires a centrifugation step to pellet bacteria present in the clinical sample. The limitation of using a centrifuge in the field can be easily overcome with the use of Paperfuge [34]. Alternative methods of sample lysis such as alkaline lysis and water lysis have been used for RPA-based detection of other microorganisms [18], and it would of interest to evaluate the compatibility of Paperfuge and various lysis methods with the current RPA-LFD assay.

Recently a RPA-based detection method has been reported for *M*. *ovipneumoniae* using 16S rRNA gene as the target [35]. However, the authors evaluated the specificity of their assay against only six mycoplasma species, the specificity of the assay requires further validation. In addition, mycoplasma species have very high sequence similarity between their 16S rRNA gene regions. Therefore, high annealing temperatures are often required for amplification of the 16S rRNA gene to detect mycoplasmas because lower temperature could lead to non-specific binding of the primers and impact the sensitivity and specificity of the assay [36, 37]. In the current study, we demonstrated the specificity of the RPA-LFD against 12 mycoplasma species and 36 other common pathogenic microorganisms of ruminants. In addition, the RPA-LFD developed in this study had a detection limit of 10 fg using genomic DNA from *M*. *ovipneumoniae*, which is equivalent to 9 genome copies per reaction, suggesting higher sensitivity of the assay compared to other reported molecular diagnostic assays [14, 35]. This detection limit was comparable to the real-time PCR assay developed for the same target gene in the present study. The clinical performance of the RPA-LFD assay developed in the study was better compared to the real-time PCR with both purified DNA (97.4% and 95.6%, respectively) and lysate (50% and 34.4%, respectively). In our study, animals (n = 25) with confirmed *M*. *ovipneumoniae* infection were tested and the results showed the same sensitivity of both the RPA-LFD and real-time enabled detection of *M*. *ovipneumoniae* in all experimentally infected animals. Although the detection rate of RPA-LFD as well as real-time PCR was found to be lower for lysate compared to purified DNA, it would be sufficient to provide the status of the infection at herd level.

Pneumonia impacts the animal health and productivity and thereby still remains one of the main causes of economic loss to the sheep industries worldwide [6, 8]. While *M*. *ovipneumoniae* are common in the upper respiratory tract of sheep, further studies are required to understand factors that drive pathogenicity. Environmental factors including high temperature along with drier weather and dustier conditions could lead to increase incidences of pneumonia in livestock [2]. Treatment for bacterial pneumonia is often based on antimicrobial therapy. While the careful use of antibiotics is advisable to treat animal diseases, overuse can contribute to antimicrobial resistance. RPA-based assays could potentially be used as screening tools on farm to define infection status of animals using nasal swabs, which are convenient to use. This will allow farmers to make informed decisions on when treatment is needed and identifying the animals requiring treatment and could reduce unnecessary use of antibiotics on farm. The results presented here showed the ability of the RPA-LFD to detect *M*. *ovipneumoniae* with better detection rate compared to the real-time PCR in clinical nasal samples without DNA extraction. Other ruminants such as big horn sheep are also affected by *M*. *ovipneumoniae* after commingling with sheep and the test described here will be useful to rule out *M*. *ovipneumoniae* infection in big horn sheep [38]. Overall, the findings indicate a new rapid and easy way to detect *M*. *ovipneumoniae* infection in clinical samples.

In summary, the results provide evidence for a sensitive and specific RPA-LFD assay to detect *M*. *ovipneumoniae*. This offers rapid and fast detection of *M*. *ovipneumoniae* in clinical samples without the need for DNA purification. These results warrant further studies to validate the assay using a larger number of clinical samples. Additionally, the study has provided proof-of-concept for the development of a novel field-applicable diagnostic tool, using the fluorescence-based assay with integration into a microfluidics platform. Such a tool could be deployed on-farm as a point-of-care diagnostic test.

## Supporting information

S1 Fig Comparison of PCR-based detection of p113 and WP_069098309.1 gene target. **A**; Gradient PCR of P113 and WP_069098309.1 gene targets. Standard PCR was performed using 10^4^ copies of standard DNA_P113 and standard DNA_WP_069098309.1 with specific primers annealed at temperature gradient of 55, 55.8, 56.9, 58.1, 59.2, 60.1, 61, 61.9, 62.8, 63.7, 64.2 and 65°C, **B**; Standard PCR of P113 and WP_069098309.1 gene targets. Standard PCR was performed using 10 ng of purified genomic DNA from *M*. *ovipneumoniae* with specific primers annealed at 55°C and 60°C. Lane; M represents 50-base pair molecular weight ladder, 1; Empty, 2; P113 at 55°C, 3; P113 at 60°C, 4; Empty, 5; WP_069098309.1 at 55°C, 6; WP_069098309.1 at 60°C. The amplification was performed for 40 cycles and after completion, amplicons were separated by agarose gel electrophoresis.
(DOCX)

S2 Fig Specificity of RPA-LFD and real-time PCR. **A; The specificity of RPA-LFD was assed using genomic DNA from common bacterial pathogens and parasites.** Lane 1 to 46, *Mycoplasma ovipneumoniae* (positive control), *Mycoplasma ovipneumoniae*-16 (field isolate), *Mycoplasma ovipneumoniae*-90 (field isolate), *Mycoplasma ovipneumoniae*-103 (field isolate), *Mycoplasma bovis* (PG45), *Mycoplasma dispar*, *Mycoplasma bovirhinis* (17D0278), *Mycoplasma bovoculi*, *Mycoplasma bovigenitalium*, *Mycoplasma canis*, *Mycoplasma gallinarum*, *Mycoplasma putrefaciens*, *Mycoplasma capricolum capripneumoniae*, *Mycoplasma mycoides* subsp. *capri*, *Mycoplasma feriruminatoris*, *Mycoplasma leachii*, *Mannheimia haemolytica*, *Pasteurella multocida*, *Staphylococcus aureus*, *Streptococcus uberus*, *Streptococcus pyogenes*, *Streptococcus agalactiae*, *Salmonella typhimurium*, *Escherichia coli* O157:H7, *Enterobacter aerogenes*, *Pseudomonas aeruginosa*, *Bacillus subtilis*, *Bacillus cereus*, *Mycobacterium bovis*, *Mycobacterium paratuberculosis* K-10, *Mycobacterium paratuberculosis* C-type (field isolate), *Mycobacterium paratuberculosis* S-type (field isolate), *Clostridium tetani*, *Clostridium perfringens* Type C, *Clostridium novyi*, *Clostridium chauvoei*, *Clostridium septicum*, *Clostridium hemolyticum*, *Trueperella pyogenes*, *Listeria monocytogenes*, *Leptospira interrogans* serovar *Hardjo*, *Klebsiella pneumoniae*, *Haemonchus contortus*, *Teladorsagia circumcincta*, *Bos taurus*, *Ovis aries*, and Lane NC: H_2_O, B, the specificity of real-time PCR was assessed against the same bacterial pathogens. Only *Mycoplasma ovipneumoniae* (positive control), *Mycoplasma ovipneumoniae*-16 (field isolate), *Mycoplasma ovipneumoniae*-90 (field isolate), *Mycoplasma ovipneumoniae*-103 (field isolate) gave positive signals and all the remaining samples were negative. The PCR products were run on 2% agarose gel with a 100 bp ladder. Lane 1–92 shows PCR product for each sample run in duplicate with H_2_O (NC) control.
(DOCX)

S3 Fig Evaluation of inhibitory effects of the lysed clinical samples on the RPA-LFD. Three different volumes 1, 2.5 or 5 μL of lysed clinical samples were used for the RPA reaction for 25 min at 39°C. Amplified dual-labelled amplicons were visualized using LFD sticks.
(DOCX)

S1 Table Source of various bacterial, parasite, bovine and ovine species used for genomic DNA isolation. (DOCX)

S2 Table *In silico* evaluation of the specificity of the RPA probes and primers against 36 genomes of pathogenic bacteria and two parasitic nematodes of ruminants as well as bovine and ovine. Fuzznuc function was used to determine specificity of the primers and probes against the genomes *in silico*. Parameters were set to examine both the strands allowing up to 10 mismatches. No complementary regions were found for primers, allowing up to 5 mismatches. Matches were found for only forward and reverse primers allowing up to 10 mismatches but were found to be minimum 10kb apart on the genomes tested. No complementary regions were found for the probe allowing up to 10 mismatches.
(DOCX)

S1 Raw images (PDF)
