# Supplementary material for: Recombinase polymerase amplification assay combined with a dipstick-readout for rapid detection of Mycoplasma ovipneumoniae infections
Source: PLoS One. 2021 Feb 4;16(2):e0246573. doi: 10.1371/journal.pone.0246573 (PMC7861559; doi:10.1371/journal.pone.0246573)
Supplement: S1 Fig — A; Gradient PCR of P113 and WP_069098309.1 gene targets. Standard PCR was performed using 104 copies of standard DNA_P113 and standard DNA_WP_069098309.1 with specific primers annealed at temperature gradient of 55, 55.8, 56.9, 58.1, 59.2, 60.1, 61, 61.9, 62.8, 63.7, 64.2 and 65°C, B; Standard PCR of P113 and WP_069098309.1 gene targets. Standard PCR was performed using 10 ng of purified genomic DNA from M. ovipneumoniae with specific primers annealed at 55°C and 60°C. Lane; M represents 50-base pair molecular weight ladder, 1; Empty, 2; P113 at 55°C, 3; P113 at 60°C, 4; Empty, 5; WP_069098309.1 at 55°C, 6; WP_069098309.1 at 60°C. The amplification was performed for 40 cycles and after completion, amplicons were separated by agarose gel electrophoresis. (DOCX) [file pone.0246573.s001.docx]

**A**


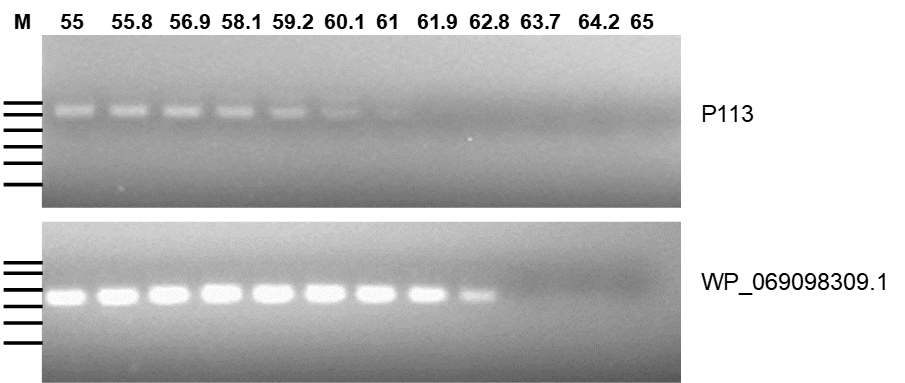


**B**


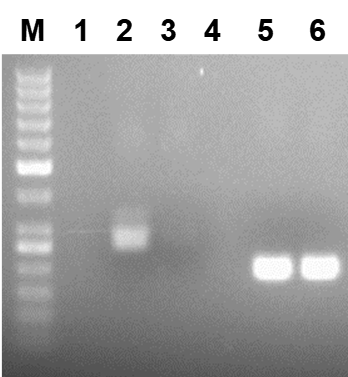


**S1 Fig.** **Comparison of PCR-based detection of p113 and WP_069098309.1 gene target.** **A**; Gradient PCR of P113 and WP_069098309.1 gene targets. Standard PCR was performed using 10^4^ copies of standard DNA_P113 and standard DNA_WP_069098309.1 with specific primers annealed at temperature gradient of 55, 55.8, 56.9, 58.1, 59.2, 60.1, 61, 61.9, 62.8, 63.7, 64.2 and 65°C, **B**; Standard PCR of P113 and WP_069098309.1 gene targets. Standard PCR was performed using 10 ng of purified genomic DNA from *M. ovipneumoniae* with specific primers annealed at 55°C and 60°C. Lane; M represents 50-base pair molecular weight ladder, 1; Empty, 2; P113 at 55°C, 3; P113 at 60°C, 4; Empty, 5; WP_069098309.1 at 55°C, 6; WP_069098309.1 at 60°C. The amplification was performed for 40 cycles and after completion, amplicons were separated by agarose gel electrophoresis.
