# Supplementary material for: Recombinase polymerase amplification assay combined with a dipstick-readout for rapid detection of Mycoplasma ovipneumoniae infections
Source: PLoS One. 2021 Feb 4;16(2):e0246573. doi: 10.1371/journal.pone.0246573 (PMC7861559; doi:10.1371/journal.pone.0246573)
Supplement: S2 Fig — A; The specificity of RPA-LFD was assed using genomic DNA from common bacterial pathogens and parasites. Lane 1 to 46, Mycoplasma ovipneumoniae (positive control), Mycoplasma ovipneumoniae-16 (field isolate), Mycoplasma ovipneumoniae-90 (field isolate), Mycoplasma ovipneumoniae-103 (field isolate), Mycoplasma bovis (PG45), Mycoplasma dispar, Mycoplasma bovirhinis (17D0278), Mycoplasma bovoculi, Mycoplasma bovigenitalium, Mycoplasma canis, Mycoplasma gallinarum, Mycoplasma putrefaciens, Mycoplasma capricolum capripneumoniae, Mycoplasma mycoides subsp. capri, Mycoplasma feriruminatoris, Mycoplasma leachii, Mannheimia haemolytica, Pasteurella multocida, Staphylococcus aureus, Streptococcus uberus, Streptococcus pyogenes, Streptococcus agalactiae, Salmonella typhimurium, Escherichia coli O157:H7, Enterobacter aerogenes, Pseudomonas aeruginosa, Bacillus subtilis, Bacillus cereus, Mycobacterium bovis, Mycobacterium paratuberculosis K-10, Mycobacterium paratuberculosis C-type (field isolate), Mycobacterium paratuberculosis S-type (field isolate), Clostridium tetani, Clostridium perfringens Type C, Clostridium novyi, Clostridium chauvoei, Clostridium septicum, Clostridium hemolyticum, Trueperella pyogenes, Listeria monocytogenes, Leptospira interrogans serovar Hardjo, Klebsiella pneumoniae, Haemonchus contortus, Teladorsagia circumcincta, Bos taurus, Ovis aries, and Lane NC: H2O, B, the specificity of real-time PCR was assessed against the same bacterial pathogens. Only Mycoplasma ovipneumoniae (positive control), Mycoplasma ovipneumoniae-16 (field isolate), Mycoplasma ovipneumoniae-90 (field isolate), Mycoplasma ovipneumoniae-103 (field isolate) gave positive signals and all the remaining samples were negative. The PCR products were run on 2% agarose gel with a 100 bp ladder. Lane 1–92 shows PCR product for each sample run in duplicate with H2O (NC) control. (DOCX) [file pone.0246573.s002.docx]

**A**


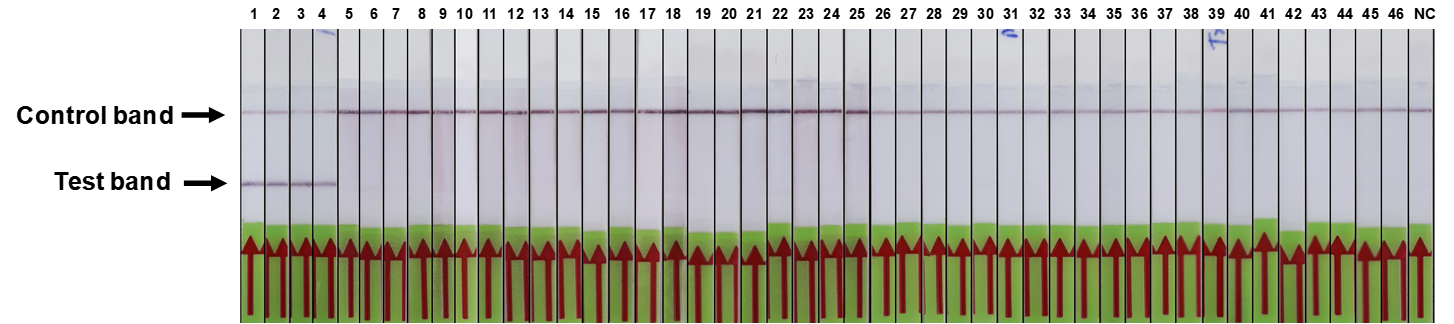


**B**


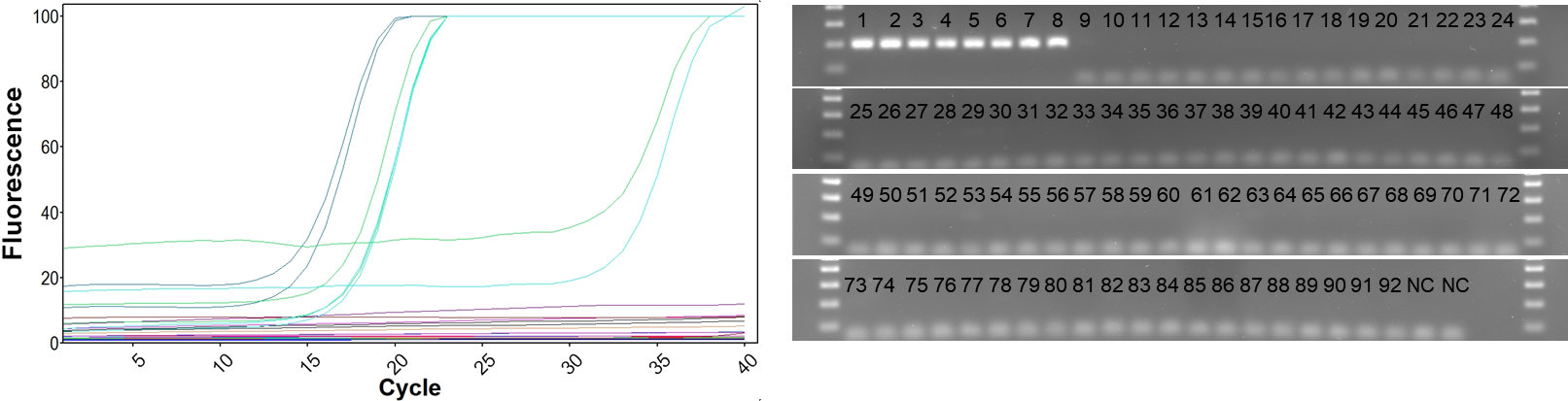


**S2 Fig**. **Specificity of RPA-LFD and real-time PCR. A; The specificity of RPA-LFD was assed using genomic DNA from common bacterial pathogens and parasites.** Lane 1 to 46, *Mycoplasma ovipneumoniae* (positive control), *Mycoplasma ovipneumoniae*-16 (field isolate), *Mycoplasma ovipneumoniae*-90 (field isolate), *Mycoplasma ovipneumoniae*-103 (field isolate), *Mycoplasma bovis* (PG45), *Mycoplasma dispar*, *Mycoplasma bovirhinis* (17D0278), *Mycoplasma bovoculi*, *Mycoplasma bovigenitalium*, *Mycoplasma canis*, *Mycoplasma gallinarum*, *Mycoplasma putrefaciens*, *Mycoplasma capricolum capripneumoniae*, *Mycoplasma mycoides* subsp. *capri*, *Mycoplasma feriruminatoris*, *Mycoplasma leachii*, *Mannheimia haemolytica*, *Pasteurella multocida*, *Staphylococcus aureus*, *Streptococcus uberus*, *Streptococcus pyogenes*, *Streptococcus agalactiae*, *Salmonella typhimurium*, *Escherichia coli* O157:H7, *Enterobacter aerogenes*, *Pseudomonas aeruginosa*, *Bacillus subtilis*, *Bacillus cereus*, *Mycobacterium bovis*, *Mycobacterium paratuberculosis* K-10, *Mycobacterium paratuberculosis* C-type (field isolate), *Mycobacterium paratuberculosis* S-type (field isolate), *Clostridium tetani*, *Clostridium perfringens* Type C, *Clostridium novyi*, *Clostridium chauvoei*, *Clostridium septicum*, *Clostridium hemolyticum*, *Trueperella pyogenes*, *Listeria monocytogenes*, *Leptospira interrogans* serovar *Hardjo*, *Klebsiella pneumoniae*, *Haemonchus contortus*, *Teladorsagia circumcincta*, *Bos taurus*, *Ovis aries*, and Lane NC: H_2_O, B, the specificity of real-time PCR was assessed against the same bacterial pathogens. Only *Mycoplasma ovipneumoniae* (positive control), *Mycoplasma ovipneumoniae*-16 (field isolate), *Mycoplasma ovipneumoniae*-90 (field isolate), *Mycoplasma ovipneumoniae*-103 (field isolate) gave positive signals and all the remaining samples were negative. The PCR products were run on 2% agarose gel with a 100 bp ladder. Lane 1-92 shows PCR product for each sample run in duplicate with H_2_O (NC) control.
