# Supplementary material for: Recombinase polymerase amplification assay combined with a dipstick-readout for rapid detection of Mycoplasma ovipneumoniae infections
Source: PLoS One. 2021 Feb 4;16(2):e0246573. doi: 10.1371/journal.pone.0246573 (PMC7861559; doi:10.1371/journal.pone.0246573)
Supplement: S3 Fig — Three different volumes 1, 2.5 or 5 μL of lysed clinical samples were used for the RPA reaction for 25 min at 39°C. Amplified dual-labelled amplicons were visualized using LFD sticks. (DOCX) [file pone.0246573.s003.docx]

**S3 Fig. Evaluation of inhibitory effects of the lysed clinical samples on the RPA-LFD.** Three different volumes 1, 2.5 or 5 µL of lysed clinical samples were used for the RPA reaction for 25 min at 39°C. Amplified dual-labelled amplicons were visualized using LFD sticks.
