# Supplementary material for: Recombinase polymerase amplification assay combined with a dipstick-readout for rapid detection of Mycoplasma ovipneumoniae infections
Source: PLoS One. 2021 Feb 4;16(2):e0246573. doi: 10.1371/journal.pone.0246573 (PMC7861559; doi:10.1371/journal.pone.0246573)
Supplement: S1 Table — (DOCX) [file pone.0246573.s004.docx]

**S1 Table**: **Source of various bacterial, parasite, bovine and ovine species used for genomic DNA isolation.**

| **Species** | **Reference** |
| --- | --- |
| *Mycoplasma ovipneumoniae* reference strain: 1959) | NZRM |
| *Mycoplasma ovipneumoniae* isolate16 | (1) |
| *Mycoplasma ovipneumoniae* isolate90 | (1) |
| *Mycoplasma ovipneumoniae* isolate103 | (1) |
| *Mycoplasma bovis* (PG45) | (2) |
| *Mycoplasma dispar* | (2) |
| *Mycoplasma bovirhinis* (17D0278) | (2) |
| *Mycoplasma bovoculi* | (2) |
| *Mycoplasma bovigenitalium* | (2) |
| *Mycoplasma canis* | (2) |
| *Mycoplasma gallinarum* | (2) |
| *Mycoplasma putrefaciens* | (2) |
| *Mycoplasma capricolum* | (2) |
| *Mycoplasma mycoides*subsp.*capri* | (2) |
| *Mycoplasma feriruminatoris* | (2) |
| *Mycoplasma leachii* | (2) |
| *Mannheimia haemolytica* | NZRM |
| *Pasteurella multocida* | NZRM |
| *Staphylococcus aureus* | NZRM |
| *Streptococcus uberus* | NZRM |
| *Streptococcus pyogenes* | NZRM |
| *Streptococcus agalactiae* | NZRM |
| *Salmonella typhimurium* | NZRM |
| *Escherichia coli* O157:H7 | NZRM |
| *Enterobacter aerogenes* | NZRM |
| *Pseudomonas aeruginosa* | NZRM |
| *Bacillus subtilis* | NZRM |
| *Bacillus cereus* | NZRM |
| *Mycobacterium bovis* | (3) |
| *Mycobacterium paratuberculosis* K-10 | ATCC BAA-968 |
| *Mycobacterium paratuberculosis* C*-*type | (4) |
| *Mycobacterium paratuberculosis* S-type | (4) |
| *Clostridium tetani* | NZRM |
| *Clostridium perfringens* Type C | NZRM |
| *Clostridium novyi* | NZRM |
| *Clostridium chauvoei* | NZRM |
| *Clostridium septicum* | NZRM |
| *Clostridium hemolyticum* | NZRM |
| *Trueperella pyogenes* | NZRM |
| *Listeria monocytogenes* | NZRM |
| *Leptospira interrogans* serovar *Hardjo* | NZRM |
| *Klebsiella pneumoniae* | NZRM |
| *Haemonchus contortus* | (5) |
| *Teladorsagia circumcincta* | (5) |
| Cow | *Bos taurus* |
| Sheep | *Ovis aries* |

**References**

1. Bridgeman B, Gupta SK, Murray A, E. A, Dukkipati R, Wedlock DN. Draft genome sequence of a New Zealand isolate of *Mycoplasma ovipneumoniae*. Microbiol Resour Announc. 2020.

2. Liljander A, Yu M, O'Brien E, Heller M, Nepper JF, Weibel DB, et al. Field-applicable recombinase polymerase amplification assay for rapid detection of *Mycoplasma capricolum* subsp. *capripneumoniae*. J Clin Microbiol. 2015;53(9):2810-5.

3. Price-Carter M, Brauning R, de Lisle GW, Livingstone P, Neill M, Sinclair J, et al. Whole genome sequencing for determining the source of *Mycobacterium bovis* infections in livestock herds and wildlife in New Zealand. Front Vet Sci. 2018;5:272.

4. Verdugo C, Pleydell E, Price-Carter M, Prattley D, Collins D, de Lisle G, et al. Molecular epidemiology of *Mycobacterium avium* subsp. *paratuberculosis* isolated from sheep, cattle and deer on New Zealand pastoral farms. Prev Vet Med. 2014;117(3-4):436-46.

5. Bisset SA, Knight JS, Bouchet CL. A multiplex PCR-based method to identify strongylid parasite larvae recovered from ovine faecal cultures and/or pasture samples. Vet Parasitol. 2014;200(1-2):117-27.
