# Supplementary material for: Recombinase polymerase amplification assay combined with a dipstick-readout for rapid detection of Mycoplasma ovipneumoniae infections
Source: PLoS One. 2021 Feb 4;16(2):e0246573. doi: 10.1371/journal.pone.0246573 (PMC7861559; doi:10.1371/journal.pone.0246573)
Supplement: S2 Table — Fuzznuc function was used to determine specificity of the primers and probes against the genomes in silico. Parameters were set to examine both the strands allowing up to 10 mismatches. No complementary regions were found for primers, allowing up to 5 mismatches. Matches were found for only forward and reverse primers allowing up to 10 mismatches but were found to be minimum 10kb apart on the genomes tested. No complementary regions were found for the probe allowing up to 10 mismatches. (DOCX) [file pone.0246573.s005.docx]

**Table S2**: ***In silico* evaluation of the specificity of the RPA probes and primers against 36 genomes of pathogenic bacteria and two parasitic nematodes of ruminants as well as bovine and ovine.** Fuzznuc function was used to determine specificity of the primers and probes against the genomes *in silico*. Parameters were set to examine both the strands allowing up to 10 mismatches. No complementary regions were found for primers, allowing up to 5 mismatches. Matches were found for only forward and reverse primers allowing up to 10 mismatches but were found to be minimum 10kb apart on the genomes tested. No complementary regions were found for the probe allowing up to 10 mismatches.

|  | **Species** | **Assembly** |
| --- | --- | --- |
| 1 | *Mycoplasma bovis* PG45 clone MU clone A2 | GCF_000183385.1_ASM18338v1 |
| 2 | *Mycoplasma dispar* strain ATCC 27140 | GCF_000941075.1_ASM94107v1 |
| 3 | *Mycoplasma bovirhinis* strain NCTC10118 | GCF_900660515.1_50766_E01-3 |
| 4 | *Mycoplasma bovoculi* M165/69 | GCF_000524555.1_ASM52455v1 |
| 5 | *Mycoplasma bovigenitalium* 51080 | GCF_000367805.1_version_1.0 |
| 6 | *Mycoplasma canis* PG 14 | GCF_001553195.1_ASM155319v1 |
| 7 | *Mycoplasma gallinarum* DSM 19816 | GCF_000621085.1_ASM62108v1 |
| 8 | *Mycoplasma putrefaciens* KS1 | GCF_000224105.1_ASM22410v1 |
| 9 | *Mycoplasma capricolum subsp. capricolum* ATCC 27343 | GCF_000012765.1_ASM1276v1 |
| 10 | *Mycoplasma mycoides*subsp.*capri* | GCF_001267815.1_ASM126781v1 |
| 11 | *Mycoplasma feriruminatoris strain* G5847 | GCF_000327395.1_ASM32739v1 |
| 12 | Mycoplasma leachii PG50 clone MU clone A8 | GCF_000183365.1_ASM18336v1 |
| 13 | Mannheimia haemolytica M42548 | GCF_000376645.1_ASM37664v1 |
| 14 | *Pasteurella multocida strain* ATCC 43137 | GCF_000754275.1_ASM75427v1 |
| 15 | *Staphylococcus aureus* subsp. *aureus* NCTC 8325 | GCF_000013425.1_ASM1342v1 |
| 16 | *Streptococcus uberis* 0140J | GCF_000009545.1_ASM954v1 |
| 17 | *Streptococcus pyogenes* M1 GAS | GCF_000006785.2_ASM678v2 |
| 18 | *Streptococcus agalactiae* 2603V/R | GCF_000007265.1_ASM726v1 |
| 19 | *Salmonella enterica* subsp. *enterica* serovar *Typhi* strain CT18 | GCF_000195995.1_ASM19599v1 |
| 20 | *Escherichia coli* O157:H7 | GCF_000005845.2_ASM584v2 |
| 21 | *Enterobacter aerogenes* KCTC 2190 | GCF_000215745.1_ASM21574v1 |
| 22 | *Pseudomonas aeruginosa* PAO1 | GCF_000006765.1_ASM676v1 |
| 23 | *Bacillus cereus* ATCC 14579 | GCF_000007825.1_ASM782v1 |
| 24 | *Bacillus subtilis* subsp. *subtilis* strain *168* | GCF_000009045.1_ASM904v1 |
| 25 | *Mycobacterium tuberculosis* H37Rv | GCF_000195955.2_ASM19595v2 |
| 26 | *Mycobacterium avium* subsp*. paratuberculosis* K-10 | GCF_000007865.1_ASM786v1 |
| 27 | *Clostridium tetani* E88 | GCF_000007625.1_ASM762v1 |
| 28 | *Clostridium perfringens* ATCC 13124 | GCF_000013285.1_ASM1328v1 |
| 29 | *Clostridium novyi* NT | GCF_000014125.1_ASM1412v1 |
| 30 | *Clostridium chauvoei* JF4335 | GCF_900168365.1_JF4335 |
| 31 | *Clostridium septicum* strain P1044 | GCF_900092375.1_PRJEB146921 |
| 32 | *Clostridium haemolyticum* NCTC 8350 | GCF_000768575.1_NCTC8350_1.0 |
| 33 | *Trueperella pyogenes* strain TP6375 | GCF_000612055.1_ASM61205v1 |
| 34 | *Listeria monocytogenes* EGD-e | GCF_000196035.1_ASM19603v1 |
| 35 | *Leptospira interrogans* serovar *Lai* strain 56601 | GCF_000092565.1_ASM9256v1 |
| 36 | *Klebsiella pneumoniae* subsp. *pneumoniae* HS11286 | GCF_000240185.1_ASM24018v2 |
| 37 | *Haemonchus contortus* | GCA_007637855.2_ASM763785v2 |
| 38 | *Teladorsagia circumcincta* | GCA_002352805.1_T_circumcincta.14.0.ec.cg.pg |
| 39 | *Bos taurus* | GCF_002263795.1_ARS-UCD1.2 |
| 40 | *Ovis aries* | GCF_002742125.1_Oar_rambouillet_v1.0 |
