# Supplementary material for: Recombinase polymerase amplification assay combined with a dipstick-readout for rapid detection of Mycoplasma ovipneumoniae infections
Source: PLoS One. 2021 Feb 4;16(2):e0246573. doi: 10.1371/journal.pone.0246573 (PMC7861559; doi:10.1371/journal.pone.0246573)
Supplement: S1 Raw images — (PDF) [file pone.0246573.s006.pdf]

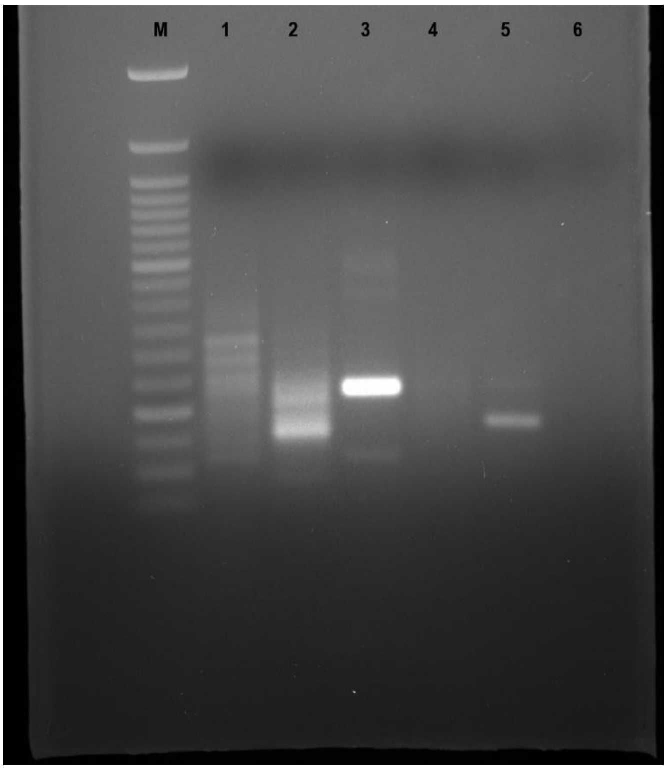

Raw image of Fig 2A. Screening of primers and nfo-probe for *Mycoplasma ovipneumoniae* RPA assay. (A) RPA amplified products using four candidate forward primers with a single biotinylated reverse primer (BR) and a probe (LFP). Lane M, represents 50-base pair molecular weight ladder, 1; F1/BR/LFP, 2; F2/BR/LFP, 3; F3/BR/LFP, 4; F4/BR/LFP, 5; positive control (supplied by Twist Amp nfo kit), 6; negative control (DNase-free water).

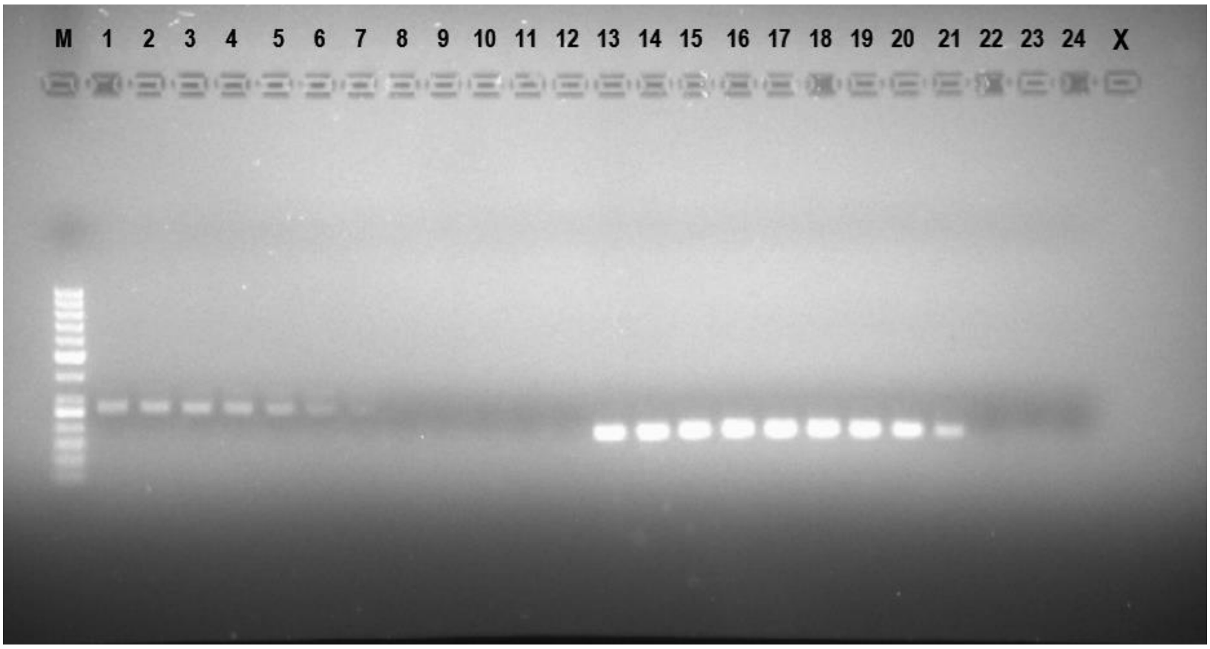

Raw image of S1A figure. Gradient PCR of P113 and WP_069098309.1 gene targets. Standard PCR was performed using 10^4 copies of standard DNA_P113 and standard DNA_WP_069098309.1 with specific primers annealed at various temperatures. Lane, M represents 50-base pair molecular weight ladder, 1-12 and 13-24; represent temperature gradient of 55, 55.8, 56.9, 58.1, 59.2, 60.1, 61, 61.9, 62.8, 63.7, 64.2 and 65°C for P113 and WP_069098309.1 genes, respectively, X; represents an empty lane. The amplification was performed for 40 cycles and after completion, amplicons were separated by agarose gel electrophoresis.

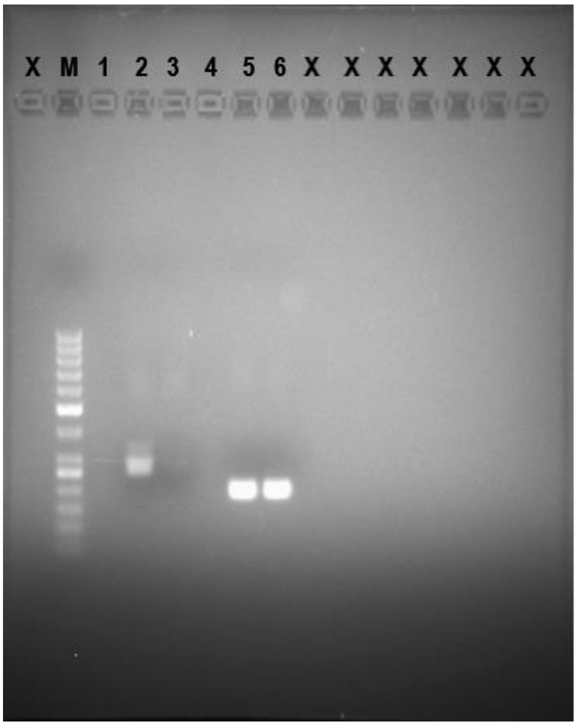

Raw image of S1B figure. Standard PCR of P113 and WP_069098309.1 gene targets. Standard PCR was performed using 10 ng of purified genomic DNA from *M. ovipneumoniae* with specific primers annealed at 55°C and 60°C. Lane; M represents 50-base pair molecular weight ladder, 1; Empty, 2; P113 at 55°C, 3; P113 at 60°C, 4; Empty, 5; WP_069098309.1 at 55°C, 6; WP_069098309.1 at 60°C, X; represents empty lanes. The amplification was performed for 40 cycles and after completion, amplicons were separated by agarose gel electrophoresis.

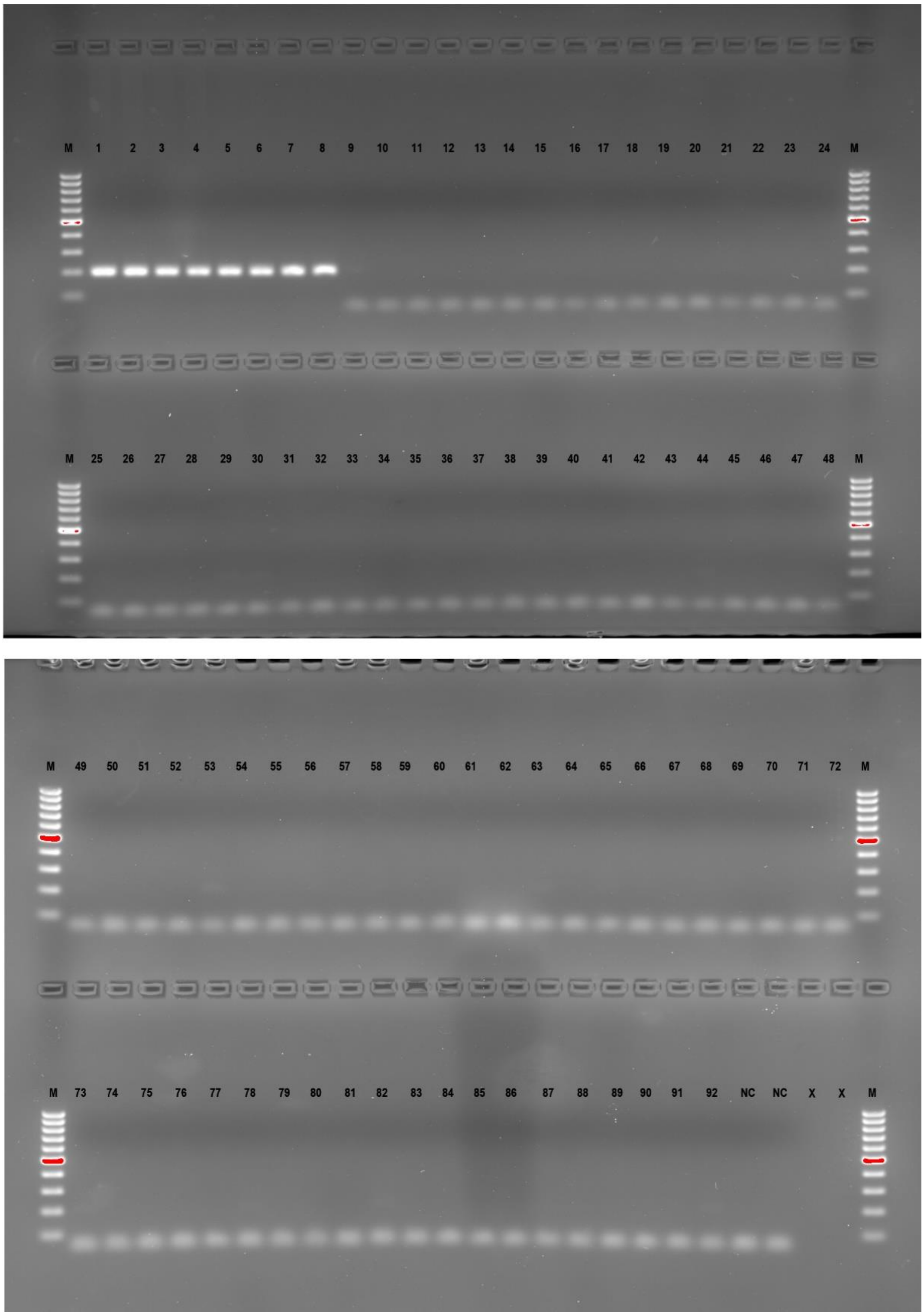

Raw image of S2B figure. Specificity of RPA-LFD and real-time PCR. A; The specificity of RPA-LFD was assed using genomic DNA from common bacterial pathogens and parasites. DNA preparations from forty-six different bacterial and parasites were subjected to real-time PCR and the PCR products were run on 2%

agarose gel. Lane M; represents 100-base pair ladder, 1-92; PCR products for each of the samples (same bacteria and parasites as given in S2 Fig) in duplicate, NC; H_2O control, X; represents empty lanes. The PCR products were separated by agarose gel electrophoresis.
